# Step-by-Step, an E-Mental Health Intervention for Depression: A Mixed Methods Pilot Study From Lebanon

**DOI:** 10.3389/fpsyt.2019.00986

**Published:** 2020-02-12

**Authors:** Melissa Joanne Harper Shehadeh, Jinane Abi Ramia, Pim Cuijpers, Rabih El Chammay, Eva Heim, Wissam Kheir, Khalid Saeed, Mark van Ommeren, Edith van’t Hof, Sarah Watts, Andreas Wenger, Edwina Zoghbi, Kenneth Carswell

**Affiliations:** ^1^Department of Mental Health and Substance Use, World Health Organization, Geneva, Switzerland; ^2^Institute of Global Health, Faculty of Medicine, University of Geneva, Geneva, Switzerland; ^3^National Mental Health Programme, Ministry of Public Health of Lebanon, Beirut, Lebanon; ^4^Department of Clinical, Neuro and Developmental Psychology, Amsterdam Public Health Research Institute, Vrije Universiteit, Amsterdam, Netherlands; ^5^Department of Psychology, University of Zurich, Zurich, Switzerland; ^6^Regional Office for the Eastern Mediterranean, World Health Organization, Cairo, Egypt; ^7^Swiss Research Institute for Public Health and Addiction, University of Zurich, Zurich, Switzerland; ^8^Country Office for Lebanon, World Health Organization, Beirut, Lebanon

**Keywords:** e-mental health, Lebanon, pilot, depression, minimally guided intervention

## Abstract

**Background:**

E-mental health is an established mode of delivering treatment for common mental disorders in many high income countries. However, evidence of its effectiveness in lower income countries is lacking. This mixed methods study presents lessons learned and preliminary data on the feasibility of a minimally guided e-mental health intervention in Lebanon. The aim was to pilot test Step-by-Step, a WHO guided e-mental health intervention, and research methods prior to future, controlled testing.

**Methods:**

Participants were recruited using social media and advertisements in primary care clinics. Participants completed baseline and post-intervention questionnaires on depression symptoms (primary outcome, PHQ-8), anxiety symptoms, well-being, disability and self-perceived problem severity, and a client satisfaction questionnaire. In addition, seven completers, four drop-outs, 11 study staff, and four clinic managers were interviewed with responses thematically analyzed. Website analytics were used to understand participant behavior when using the website.

**Results:**

A total of 129 participants signed up *via* the Step-by-Step website. Seventy-four participants started session 1 after completing pre-test questionnaires and 26 completed both baseline and post-intervention data. Among those who completed post-assessments, depression symptoms improved (PHQ-8 scores (t=5.62, p < 0.001 two-tailed, df = 25). Wilcoxon signed ranks tests showed a significant difference between baseline and post-Step-by-Step scores on all secondary outcome measures. Client satisfaction data was positive. Interview responses suggested that the intervention could be made more appropriate for younger, single people, more motivating, and easier to use. Those who utilized the support element of the intervention were happy with their relationship with the non-specialist support person (e-helper), though some participants would have preferred specialist support. E-helpers would have liked more training on complex cases. Website analytics showed that many users dropped out before intervention start, and that some re-entered screening data having been excluded from the study.

**Conclusion:**

Step-by-Step skills and techniques, model of service integration, and its non-specialist support element are acceptable. Though the sample was small and non-controlled and drop-out was high, results suggest that it may be effective in reducing depression and anxiety symptoms and increasing well-being. Lessons learned will inform content revision, the development of an app version of Step-by-Step, and the research methodology of upcoming effectiveness studies.

## Introduction

The evidence for the efficacy of e-mental health is so compelling that e-mental health programs have been included in a number of countries’ national mental health strategies and treatment guidelines, including the Netherlands, the United Kingdom, Australia, New Zealand, Sweden (1), and, since 2015, Lebanon (2). However, very little of the evidence supporting e-mental health comes from low- and middle-income countries (3, 4) or conflict or post-conflict settings.

E-mental health programs can be unguided and fully automated, or can be guided, whereby the user receives written, telephone, or face-to-face support from a health worker or trained layperson. Systematic reviews of the comparative effectiveness of guided versus unguided e-mental health interventions show that effects are higher when an intervention is guided, for example d = 0.78 guided versus d = 0.36 unguided (5) and that the support can be beneficial regardless of the qualifications of the supporter or the type of support offered (5, 6). WHO treatment guidelines for depression recognize the potential of guided and unguided self-help to vastly improve treatment coverage at a public health level (7).

Lebanon is a middle-income country in the Middle East with a long history of complex political turmoil and conflict. Civil war, border tensions, war with neighboring countries, and the conflict in Syria have all deeply impacted Lebanon and its health system. The United Nations High Commissioner for Refugees (8) reports more than 1.5 million registered Syrian refugees and many more undocumented persons of concern residing in Lebanon, further stretching Lebanon’s already limited resources.

A national representative survey (n = 2857) conducted in Lebanon before the Syrian conflict showed that one in six people met criteria for at least one mental disorder, with 27.0% of these classified as “serious” and of those with a mental disorder, just one in nine (11%) had ever obtained any treatment (9). It is to be expected that this situation has worsened since the survey was conducted, especially considering the more recent influx of displaced persons affected by the conflict in Syria.

Ministry of Public Health (MoPH) data from 2015 highlighted that the main problems in accessibility to mental health services are physical and financial, with mental health services being centered in urban areas and mostly in the private sector at an elevated out-of-pocket cost. The MoPH has worked extensively with the support of WHO on reforming this sector by integrating mental health care into its network of public Primary Health Care Centers (PHCC). Nevertheless, the lack of stable financing, the lack of services in rural areas, and the prevalent stigma against mental health (10) remain the major barriers to accessing care. This all contributes to the MoPH-estimated mental health treatment gap of 90% in Lebanon and highlights the importance of a mental health intervention that can be delivered widely despite the limited human resources, limited funding, and stigmatizing beliefs that currently reduce access to care.

A substantial proportion of the population in Lebanon has regular access to mobile phones (81%) and the Internet (76%) (11). With regard to Syrian refugees in Lebanon, 100% have 3G coverage (8). These data, coupled with high literacy rates and high need mean that e-mental health could represent an important opportunity to overcome cost, stigma, and other access issues in mental health care provision to increase access to evidence based interventions. Investigating e-mental health as a service delivery strategy has been included in the MoPH Mental Health Strategy for Lebanon 2015–2020, currently under implementation by the National Mental Health Programme at MoPH.

The MoPH in Lebanon has partnered with the World Health Organization (WHO) to test a WHO digital intervention called “Step-by-Step” (SbS) (12). SbS is a brief, minimally guided self-help program for people with depression (see description below). The aim of this paper is to present the results of a preliminary, uncontrolled pilot study on a website version of SbS, including information on other elements of the process such as web analytics data from the website.

## Methods

### Pilot Study

We assessed SbS in a small uncontrolled pilot study with the aim of informing further development of the intervention and to inform the design of randomized controlled trials (RCT). In addition to collecting pre- and post-measures, we also conducted a qualitative process evaluation.

#### Intervention

SbS (Khoutweh-Khoutweh in Arabic) is a five session online intervention using behavioral activation and stress management techniques designed to ameliorate symptoms of depression (12). It has been designed as a scalable self-help intervention, which may or may not be coupled with guidance from a health worker or other helper. SbS can be accessed through a website using a smartphone, tablet, or computer. It is presented using a narrated story with interactive exercises to apply the techniques learned through the story to the users’ own life. SbS was systematically translated and adapted to the Lebanese context, the details of which can be found in a separate paper (13). More detailed information about the intervention can be found in Carswell et al., (12).

Potential participants (see below) visited the website (either in English or in Arabic), where a short recruitment video and a how-to sign-up video could be found. Users would find out if SbS could be suitable for them by completing the PHQ-9 screening questionnaire. Upon completing screening and meeting inclusion criteria, participants could set-up an account, view all study information, and complete the informed consent procedure, with the possibility to contact study staff with questions or problems signing up. After consenting, they were taken to a brief introduction session where a story character presented each baseline measure for the participant to fill in. Baseline assessment was followed by a brief exercise to identify personal strengths and a relaxation exercise, in case participants felt anxious about their symptoms due to completing the pre-assessment questionnaires. They were then able to start the intervention 3 days later, with sessions being released every 4 days. Participants had 8 weeks to complete the subsequent five sessions of SbS before being prompted to fill in post-assessments. Tablets were placed in private rooms in five of the participating PHCCs in order to provide access to those who may not own a smartphone or have access to the Internet.

Throughout the intervention, trained psychology and public health graduates (referred to as “e-helpers”) could offer minimal assistance to SbS users (if desired by the user) through 15–20 minutes of weekly phone calls or email messaging. This assistance included technical website support, motivational support, and clinical support such as simple problem solving and troubleshooting SbS exercises. E-helpers were non-specialists trained over 6 days by study staff on the intervention as well as on mental health conditions, communication skills, and working with distressed people. They received group clinical and management supervision every 2 weeks, as well as on-demand telephone clinical supervision with a clinical psychologist. Users did not have to accept e-helper support in order to use SbS, but they were encouraged to do so.

#### Study Population and Recruitment

The target population included male and female Lebanese, Palestinian, and Syrian participants who self-reported as over the age of 18, were resident in Lebanon, scored above a locally established cutoff of 10 or above on the PHQ-9 (14), and were not at risk of suicide (according to item nine on the PHQ-9 screening questionnaire and two follow-up questions). Participants provided informed consent as part of the sign-up procedure.

Recruitment began in October 2017 through seven government-supported PHCC across the country and two Beirut private family health clinics. Posters and leaflets were placed in waiting rooms with a short video where screens were available and a trained focal point at each center provided information about the study. We also conducted two social media advertisement campaigns through the MoPH Facebook network and posted advertisements and recruitment links on two Beirut University Facebook and WhatsApp groups. Recruitment took place over 6 months using non-stigmatizing language in any recruitment materials with images showing people from ambiguous socio-economic status (SES) and religious backgrounds.

#### Outcome Measures

The primary outcome of the pilot study was depression symptoms measured using the PHQ-8 (14, 15), which was also repeated at the beginning of each SbS session. The PHQ-8 contains the same items as the study’s screener, the PHQ-9, minus the item on suicide ideation. Secondary outcome measures were the WHO Disability Assessment Scale (WHODAS) 2.0 (16), General Anxiety Disorder seven (GAD-7) (14, 17), WHO-five wellbeing index (WHO5) (18), and the Psychological Outcomes Profile questionnaire (PSYCHLOPS) (19), a self-report problem rating scale. We also provided a satisfaction questionnaire at post-assessment which was adapted from the Client Satisfaction Questionnaire (20).

For ethical reasons, participants could skip a question if they did not want to answer it. This led to some missing values, which were imputed by taking the mean of the other item responses of the measure and rounding this down or up to the nearest integer.

#### Analysis

Data was collected by the web program and downloaded into Stata version 14 and into Comprehensive Meta-Analysis version 3.3.070. Missing data values were imputed for post-intervention PHQ-8 completers only (given the analysis was per-protocol). As an example, for the primary outcome measure at pre-intervention (N = 26), zero out of 208 item responses were imputed and at post-intervention, 3/208 item responses were imputed (1.4%). For other measures, imputation rates were similarly low. Descriptive statistics and simple tests of statistical differences were run. Despite the study not being powered to detect effect sizes, an effect size calculation for completers gave a crude idea of the magnitude of the effects of completing SbS. Effect size was measured as the difference between the means divided by the pooled standard deviation and adjusted for the correlation between pre- and post-intervention total PHQ-8 score.

When checking for skewness and kurtosis of the small data set of post-intervention assessment completers only, data was found to be right-skewed for all measures apart from the PHQ-8 either at pre- and/or post-assessment. We therefore carried out a t-test and effect size calculation on PHQ-8 data and Wilcoxon matched pairs signed ranks tests on the other outcome measures.

### Process Evaluation and Web Analytics Analysis

The process evaluation comprised 1-hour long interviews with 11 participants in the study (seven of these had completed the intervention and four had dropped out, i.e. completed less than four sessions). Study participants were asked during their support sessions or in the PHCC if they wanted to participate in a telephone or face-to-face interview after the study (face-to-face interviewees were offered 10 USD to cover travel expenses). Of those who had accepted, 11 were selected at random toward the end of the study period. All participants provided verbal informed consent on the phone prior to the interviews. Participants were diverse with regards to nationality, gender, marital status, and area of residence. Interviews were conducted by phone or face-to-face, depending on the preference of the participants. Those who attended the face to face interviews were remunerated 10$ for transportation. In addition to interviews with participants, interviews were conducted face-to-face with the four e-helpers, the clinical supervisor, six front line workers from several PHCCs, and four directors of PHCCs.

A semi-structured interview schedule was used to guide the discussion with participants. This schedule included questions on five main themes: overall experience, intervention content, rapport with e-helpers, intervention adherence, and burden of assessments (see **Supplementary Data Sheet 1** for Process evaluation semi-structured interview guide for participants of the intervention). Interviews were recorded and transcribed. Interviews were carried out and analyzed by the local project coordinator (JAR) and a consultant research assistant (recruited to carry out some of the interviews with project staff in order to minimize bias in responses).

Interview data (in the form of notes) was recorded, transcribed, translated into English, and thematically analyzed. Responses were categorized into themes by one of the two data collectors and a third team member checked the thematic coding against the original recordings or notes of the interviews (EH). Discrepancies in thematic coding of information were discussed between data collector and the data checker.

Available website analytics were compiled using the Matomo (Piwik) web analytics application V3.3.0, including information about the number of visits to the site, descriptive data on the number of sessions completed by participants, weekly depression scores, and number of participants who dropped-out.

## Results

### Demographic and Usage Data

Out of 149 applicants, 129 completed the baseline assessment and met inclusion criteria (of which 100 were female). Their mean age was 27.7 years old (range 18–70). Demographic characteristics are displayed in **Table 1**.

**Table 1:** Characteristics of study participants who chose to provide demographic information.

Demographic characteristic	Frequency
Nationality (n = 126)	
Lebanese	111 (88%)
Palestinian	8 (6%)
Syrian	5 (4%)
Other	2 (2%)
Gender (n = 129)	
Female	100 (78%)
Male	29 (22%)
Highest level of education (n = 124)	
Primary school (3–6 y)	1 (1%)
Elementary (6–14 y)	3 (2%)
Secondary school (15–17 y)	14 (11%)
University (18+ y)	97 (78%)
Technical school (18+ y)	9 (7%)
Occupation (n = 128)	
Employed paid	44 (34%)
Non-paid work	2 (2%)
Student	41 (32%)
Homemaker	12 (9%)
Unemployed	23 (18%)
Other	6 (5%)
Location (n = 127)	
Beirut	54 (43%)
Outside Beirut	65 (51%)
Outside Lebanon	8 (6%)
Marital status (n = 123)	
Never married	75 (61%)
Currently married	35 (28%)
Separated	6 (5%)
Divorced	6 (5%)
Widowed	1 (1%)

We had envisaged to recruit 200 people in 6 months, but we struggled to recruit this number of participants, with particularly low numbers of non-Lebanese nationals (5/126 Syrians and 8/126 Palestinians). Of the 126 people who responded when asked “how did you find out about Step-by-Step”, 81 stated they heard about SbS *via* the web (Facebook being the only website we recruited through), and 20/126 had heard about it through a friend. Twenty people selected *via* a health center (either from a health worker or by the flyers and posters placed in health centers). Of the Palestinians who did sign up, all eight were recruited in the health center of a refugee camp.

**Figure 1:**
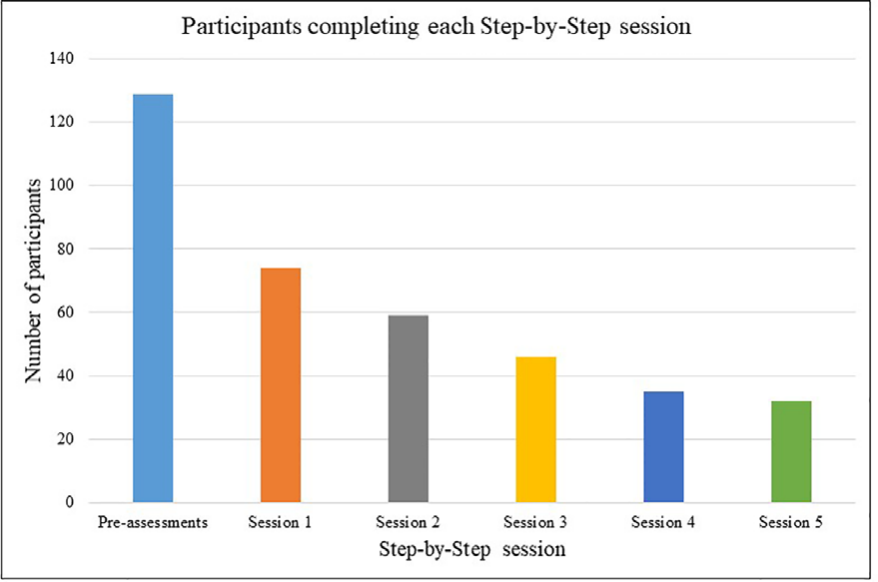
Number of participants completing Step-by-Step (SbS) sessions.

**Figure 1** shows the number of participants that completed each session of the intervention. Fifty-five (43%) of the 129 participants did not start session one after they had signed up and completed baseline assessments. Twenty-six of those 74 (35%) participants who started SbS completed post-treatment questionnaires. Thirty-two of 74 participants (43%) who started completed all five sessions and 35 (47%) completed between one and four sessions. All but three people who reached session four went on to complete the intervention. If we consider a completer to be anyone who started session 1 and completed at least four of the six sessions (n = 35), SbS had a drop-out rate of approximately 50%.

Characteristics of those participants who signed up but never returned to the Step-by Step website site were broadly similar to those who reached session four or five (completers). A two-tailed t-test showed that there was no difference in the pre-intervention PHQ-8 scores of those who signed up and did not complete, versus those who completed post-assessment (t = 0.104, p = 0.92, two-tailed, df = 217). The two groups slightly differed with regards to marital status (a larger proportion of completers were never married) and how they heard about SbS (completers being more likely to have heard about SbS through the Internet as opposed to through a friend or family member). See **Supplementary Table 1** for more information on characteristics of completers versus non-starters.

### Outcomes of Sbs

**Figure 2:**
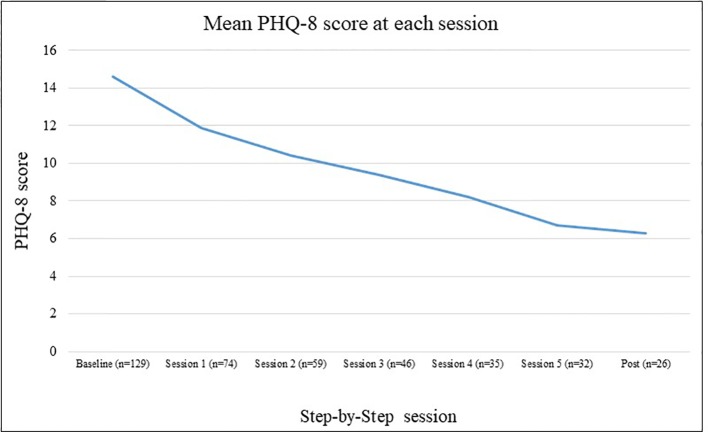
Mean Patient Health Questionnaire depression scale (PHQ-8) scores by session for all participants.

**Figure 3:**
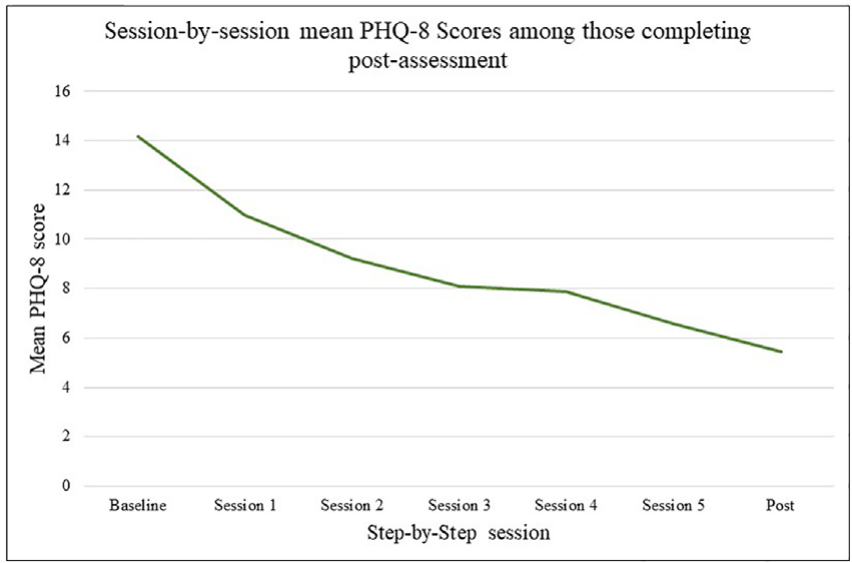
Mean Patient Health Questionnaire depression scale (PHQ-8) scores by session for those completing post assessment only.

Depression as assessed by mean PHQ-8 total scores decreased at each measurement each session among all participants and among those completing post-assessment only, as shown in **Figures 2** and **3**.

Among the 26 people who completed post intervention assessment, the mean number of sessions completed was 4.7. Mean scores on all measures decreased from baseline to post-SbS, as shown in **Table 2**. There was a significant difference between baseline and post SbS PHQ-8 scores (t = 5.62, p = < 0.01 two-tailed, df = 25). An effect size calculation (Hedges’ g) estimated the magnitude of that pre-post difference at 1.56 (95% confidence interval 1.05–2.07).

**Table 2:** Mean scores, standard deviations and results of Wilcoxon signed ranks test for all outcome measures among participants who completed post-SbS assessment.

Measure	Baseline mean and standard deviation (N = 26)	Post-SbS mean and standard deviation	Post-SbS assessment N	Z	p
**PHQ-8**	13.96 (4.75)	6.31 (5.73)	26	3.903	< 0.001
**WHODAS 2.0**	15.15 (8.37)	9.61 (9.88)	23	2.680	< 0.01
**WHO-5**	7.96 (3.30)	13.69 (5.19)	23	-3.594	< 0.001
**GAD-7**	14.11 (4.21)	7.22 (4.31)	23	3.763	< 0.001
**PSYCHLOPS**	15.85 (3.29)	6.43 (3.98)	21	3.982	< 0.001

PHQ-8, Patient Health Questionnaire depression scale; WHODAS 2.0, WHO Disability Assessment Schedule; WHO-5, WHO-Five well-being Index; GAD-7, Generalized Anxiety Disorder scale; PSYCHLOPS, Psychological Problems and Outcomes, CI, Confidence Interval.

The results from the Wilcoxon signed ranks tests showed a significant difference between baseline and post-SbS scores on all secondary outcome measures (see **Table 2**).

Despite the inclusion criteria to have scored 10 or higher on the PHQ-9 screener [a locally established cut-off, (14)], 6/26 did not indicate caseness for major depressive disorder when they completed the PHQ-8 measure at baseline shortly after screening [a score of 10 or over when using the PHQ-8, (21)]. Of these six people who did not have major depression at baseline, five had improved symptom scores and one did not change after completing SbS. Among the remaining 20 participants who had major depressive disorder at baseline (according to the PHQ-8), 75% (15/20) scored below the cut off for major depressive disorder at post assessment. Of those who remained above cut off (PHQ-8 > 9) at post-intervention, one had improved symptom scores, one had not changed and three had deteriorated (with change scores of −7, −4, and −3).

The client satisfaction questionnaire was filled in by 21 participants. Questions used a Likert scale from 1 to 5, with higher values indicating higher satisfaction. Mean item satisfaction for SbS ranged from 2.9 out of 5 (for the question “to what extent has our program met your needs?”) to 3.8 out of 5 (for the question “If a friend were in need of similar help, would you recommend our program to him or her?). The mean total satisfaction score was 26.5 out of a possible 35 indicating on average these participants indicated high levels of satisfaction.

### Process Evaluation

**Figure 4:**
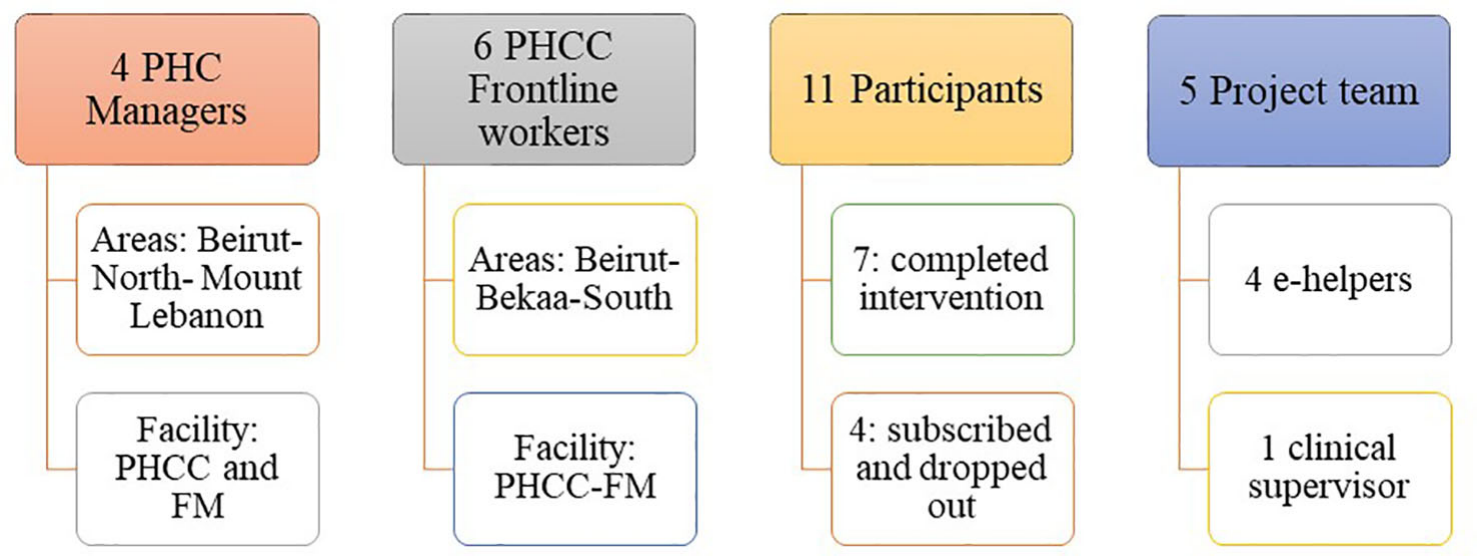
Process evaluation participants schema.

The process evaluation consisted of interviews with four clinic managers, six frontline workers, 11 participants (completers or drop-outs), the four e-helpers, and the clinical supervisor (see **Figure 4** for a visual representation).

Results revolved around five themes (which were led by the semi-structured interview questions): overall experience, intervention content, rapport with e-helpers, intervention adherence, and burden of assessments.

#### Overall Experience

##### General Perception of E-Mental Health

In general, the SbS intervention was valued and considered innovative among PHCC managers and focal persons, yet, with an acknowledgement that such an intervention needs time to be accepted by the community.

“Even the international visitors were surprised by the posters and were astonished that MoPH is leading on such advanced methods. We need much more time to reach this stage (where people are ready to use it).” (manager)

One manager expressed that she trusted face-to-face therapy only. Most frontline workers and managers agreed that the intervention is more relevant and useful to people of moderate to high SES levels and not to refugees or the disadvantaged population that they serve in their centers. The reasons cited revolved around Internet access problems, illiteracy, lack of community knowledge about mental health and the lack of time, financial and human resources to support, follow-up, and track patients.

“This intervention could be relevant to people from higher social classes; but at the PHCC level and the refugees’ level, it’s difficult cause they are mostly illiterate and don’t have the concept of caring for their mental health. Maybe better for educated people who are more aware about mental wellbeing.”

Their recommendations for service integration were to:

- Ensure a good Internet connection;
- Train and allocate assistance for SbS use;
- Conduct awareness sessions frequently and repetitively to spread awareness and normalize use of the intervention.

One frontline worker reflected on the importance of the MoPH endorsing SbS for its credibility, whereas two others expressed their willingness to be more involved and to be given the autonomy to call users and run awareness sessions and workshops by themselves:

“I wish I could be given more authority or responsibility in this, to be able to spread the word in several settings; for example, I can conduct awareness sessions and workshops without getting millions of approvals every time. The system is very bureaucratic here and is obstructing the fast pace of the recruitment in SbS.”

Frontline workers reported mixed reactions among clinic users when seeing SbS advertisements: some took pictures and were interested, while others avoided engagement or didn’t feel involved. A common finding was that the registration process was complex and inhibited the participation of many. This was echoed in the responses from SbS drop-outs, with their main reasons for not completing SbS being technical and not related to the content of the intervention.

In contrast to these reports, feedback from the seven completers was very positive. SbS was perceived as a feasible solution to physical and financial inaccessibility of services, stigma and isolation, or a great complement to the therapy that some were already receiving:

“I am a very busy person and I live far away and don’t have access to PHCC. I learned to help myself, I learned new skills to do daily. I knew about it from Facebook and I’m so glad I found it because it benefited me a lot.”

One participant even noted a drastic change in her perception and the self-stigma she had toward mental health:

“I was shocked because I didn’t want to seem “crazy”, yet, when I did it, I understood that having issues does not mean you’re crazy and that everyone might be suffering from something eventually.”

The e-helpers all reported to be happy to have taken part, for example, one described the experience as “great, unique and amazing and beneficial”. Nevertheless, half of them perceived it as useful to complement face to face therapy.

Recruitment methods could be improved, according to one completer and most e-helpers and focal persons:

“The only area of improvement is that you did not advertise for it! I saw it in a clinic and when I went back home, I searched everywhere on the Internet and couldn’t find it; so I had to go back to the clinic and look for it.”

#### Intervention Content

##### Story

Both drop-outs and completers liked the story, its pace and length (some did cite that activities were a bit difficult toward the end) and related to the symptoms of the character. Feedback was divided into two: those who were married liked the character profile while those who were not felt that it was more tailored toward people with families, impeding their ability to relate to the story. They suggested new problems and character lifestyles to be added to the story:

“I have three kids; I liked the family concept and that it reminded us about the values of the family and the fact that if I get better, my family will be better as well.” (drop-out 1)

“I liked the story, it’s from the local culture and stems from daily life situations that we are actually living. It would be nice to have more [suggestions for] activities.” (drop-out 2)

“I could relate to the symptoms but not to the lifestyle; she’s a housewife with kids while I’m a student; add examples for younger or older persons (fitting in, peer pressure, university, work)”. (completer 1)

“Instead of having the person not wanting to go to work, focus more on a situation where the person still goes to work but feels frustrated, demotivated and irritable.” (completer 2)

They also suggested to change the voice-over in the story video as it was cold and monotonous in many instances. Suggestions were made to use more lively colors and videos in English as well as in Arabic.

##### Website

In general, all participants mentioned that navigating the site was simple, yet, the log-in process was long and complicated. In fact, three of the four people who dropped out said it was partly due to technical problems or complexity of sign-up.

##### Rapport With E-Helpers

It was noted among participants interviewed and the e-helpers that there was a general preference from the public to have specialist support. Of the four drop-outs that were interviewed, three would have preferred if the e-helpers were qualified specialist therapists. One of the completers also commented on a preference for specialist support.

“When the e-helper told me they were not a psychologist, I felt “oups” as I had different expectations; if the e-helpers were experts, their words would have given them more credibility. E-helper was doing the job well but I mean e-helper was not a specialist, so they wouldn’t be able to dig more into problems or therapeutic techniques.”

“If I were to receive calls, I would have liked for someone professional to call me.”

This was confirmed by the e-helpers who stated that they struggled clarifying to participants that they were not professionals, especially to those who expected to get professional support.

“The concept of self-help is new which could explain their expectations” (e-helper)

Having said this, the three that had contact with an e-helper all commented very positively on their relationship with the e-helper and found the support positive and helpful.

“I used to wait eagerly for Monday calls to let it out in a healthy way instead of hitting my children. So really this was very helpful.”

“I didn’t feel she was a therapist, rather, she cared as a friend and wanted to make me feel better; she was compassionate. I loved it. She made me feel alive and that I exist.”

“I think that’s what makes the program very special. It gives you motivation- it makes it more personal”

From the e-helpers’ perspective, they described their rapport with the participants as very diverse and classified the users into four groups: the active and responsive users, who benefited the most from the program; the inactive yet responsive users, who received support without doing the sessions; the active yet unresponsive users, who did the sessions independently and did not wish to have support from e-helpers; and those who were both inactive and unresponsive after sign up.

In addition to the above, e-helpers shared some challenges encountered while dealing with special cases such as users who got attached to them, asked them personal questions, or were high risk cases (mainly presenting with suicidal ideation).

“Some asked me “do you love me?” others inquired about my personal information “family name, age, religion, area of origin” and one or two asked for a meet up with some of us” (e-helper)

The main recommendations regarding e-helper support were to:

- Focus more on the suicide risk management procedure in the training;
- Add more self-care techniques for the e-helpers who are susceptible to stress or burnout;
- Lengthen the shift times of the e-helpers in order to ensure cover of busy afternoon hours;
- Add notifications and reminders for participants to comply with the intervention

All these recommendations were taken into consideration by the project team in planning the next phase of the project.

##### Intervention Adherence

Problems with reminders and motivation were prevalent among participants whereby most forgot to log-in, practice their activities, and respond to the e-helpers’ messages/calls. Recommendations were made to make the website more interactive and dynamic. Two completers suggested to add push notifications and empowering messages, including praise and potentially personalized motivational messages.

E-helpers affirmed these findings by stating that they were frustrated with following up and reminding the unresponsive or the inactive users to log in, attend their support sessions or do their sessions and activities. They also recommended to add pop up notifications and reminders prior to the support sessions, and whenever a new session is available.

One drop-out stated that she lacked the motivation and time to practice the activities which added more stress on her:

“When I saw this program, I thought to myself: “that’s it! This is what’s missing in my life and I’ve got to do it!” but then I lacked motivation … I don’t have a minute to calm down and I usually stay late at work. So when I come back home, I stop doing anything- and I stress out for not being able to do the activities that I wrote. I need a break but I can’t do anything else, which makes me hate myself.”

#### Website User Behavior and Preferences

A total of 4209 unique visitors came to the site during the recruitment period, including study staff. Most people accessed the website using smartphones (62%), followed by computers (26.5%) and other devices such as tablets (12%). At sign up, participants most commonly chose to communicate to e-helpers by email or WhatsApp messaging (n = 74 or 57.4%), then by phone (n = 32 or 24.8%), and then by using the instant message “Chat” function within the website (n = 23 or 17.8%).

The analysis of web traffic provided limited but some relevant information including when there were peaks in the number of site visits, which coincided with Facebook advertising drives (December 2017 and February 2018). It also showed that between 23% and 32% of website visitors were lost at the point of screening. Between 53% (English website) and 69% (Arabic website) of people who did complete the screening repeated it if they were excluded. The web analytics showed that just 19% of the visitors (on the Arabic and English website) clicked on the detailed study information. Participants who watched the videos of the sessions watched between 21% and 75% of the video length on average.

## Discussion

The data from this uncontrolled pilot test (post intervention assessment completers n = 26) showed mean depression symptom scores to decrease at each time point throughout the intervention among those who continued the intervention. Despite a substantial attrition between baseline and post-intervention testing, session data seemingly showed symptoms to improve. Though drop-out was high, pre- and post-intervention data showed a statistically significant reduction in symptoms of depression and anxiety, improvement in wellbeing and in functioning and a reduction in the magnitude of self-defined problems pre- and post-SbS. Though the effect size calculation on depression scores should be taken with caution due to few participants, high attrition and no control group, its magnitude was great, at g = 1.56 (95% confidence interval 1.05–2.07). The uncontrolled study design and lack of post-intervention data from the numerous drop-outs means that one cannot rule out natural recovery or other extraneous variables causing this effect. Qualitative data gathered through process evaluation interviews with a number of stakeholders suggest that the intervention itself is acceptable and feasible and that motivation and/or technical problems were key reasons for drop-out, as opposed to intervention content or the general concept of the delivery model. This research shows that there are a number of considerations to incorporate into the intervention itself and into the design of further effectiveness testing.

Disproportionately few people who signed up to this study were Syrian or Palestinian. The social media campaign was limited to advertising through the MoPH Facebook pages, so was more likely to have attracted nationals who have an interest in health. Furthermore, WhatsApp advertising was limited to two university student groups, attracting younger, more affluent Lebanese students (as Syrians and Palestinians in Lebanon are less likely to go to university than Lebanese nationals). In e-mental health studies focused on Turkish and Arab migrants in the Netherlands and Australia, focused Facebook advertising was found to be an effective strategy for recruitment (22, 23). Further testing of SbS will focus more on recruiting Syrians and Palestinians as well as Lebanese people, weighing more heavily on using social media in a focused manner to capture potential participant’s attention, as well as other traditional recruitment methods. An important learning point was that recruitment materials should be tailored for different population groups and should use a broader range of social media channels. The research team is currently linking to international organizations and non-governmental organisations (NGO) that serve Syrian refugees specifically for recruitment support.

The results from this pilot study are informing an updated smartphone app and website version (hybrid) of SbS, which will include offline capabilities and features such as notifications and a within app communication channel for support. Demographic data and user feedback has also informed content changes (making SbS more tailored to unmarried, economically active users). It is expected that the new app version will address the many comments gathered in the interviews, making it easier to use and more equitable with regards to data costs for participants. Planning has started for the next phase of testing, with much more reliance on social media and other digital channels for recruitment and also calling on partner agencies, service providers, and other community resources to attract potential participants.

Though the primary effect size calculation found in this study is likely overestimated due to design limitations, it (Hedges g = 1.56) is comparative to the effect sizes shown in other studies which used guided e-mental health in the treatment of depression in high income settings (21), effect size = 0.98; (22, 23). Forthcoming RCTs will shed more light on SbS effectiveness compared to like interventions.

As an uncontrolled pilot, this study carries a number of limitations. Of the 129 people that were recruited, those completing four sessions or 80% of the intervention (considered a completer) were 35 (27%) and those completing post assessment were only 26 (20%). These figures are high compared to other e-mental health studies, which often have rates of attrition at around 33% (24). Having said this, a study on e-mental health for depression among Turkish migrants in the Netherlands had an attrition rate of 42% (post-intervention) and 62% at 4 months follow up (25).

In this study, 41% of those who signed up did not commence SbS (also a problem in other e-mental health studies, for example, Watts et al., 2013, where 33% were lost at sign-up (26). With so few participants providing both baseline and post-intervention data and without a control group, the findings should be interpreted with caution and considered only as an indication that the intervention might be efficacious. Further testing is planned for 2019 and 2020, during which we will focus recruitment activities on the seemingly more effective method of advertising on social media, additionally targeting Syrian refugees through refugee- dedicated services.

Web analytics data showed that a number of participants re-entered data presumably in order to get access to SbS after they had been excluded. This presumption is supported by the fact that 3/26 participants who completed baseline and post-SbS assessments did not indicate caseness for mild depression when they completed the PHQ-8 measure at baseline shortly after they were indicated for caseness at screening. During further testing, it will be important that website visitors cannot re-enter screening information once they have been excluded from the study.

We cannot be sure from the data gathered in the process evaluation why there was such a high number of non-starters (41%), incremental drop-out, and people who did not complete the post-assessment, though motivation was commonly mentioned by the participants we gathered feedback from. Participants were sent up to three email reminders when they did not log into the system and when their post-assessment was due. E-helpers also tried to call (up to three attempts) those who provided their phone number to remind them of the post-assessment. It seems from formative work carried out that email is not the best means of communication with the target user group as email addresses or login details are often forgotten. E-helpers recalled that the success rate (number of call attempts versus number of calls answered) of the reminder calls for post-assessment was low.

Though “non-use” attrition (i.e. people registering but never using the intervention) is not unusual in mental health interventions, we gathered anecdotal knowledge that professionals (e.g. academics, health staff and NGO staff) had signed up to the intervention out of curiosity having seen social media posts or hearing about it through colleagues. Furthermore, analytics data suggested that some users who were excluded re-submitted answers to the screener, meaning that people for whom the intervention was not designed were signing up. For example, people not suffering from depression, people having suicidal thoughts, therefore needing more targeted support or those not intending to use the intervention may have registered for SbS. This was a very important learning point and for the app version of SbS, we will need to make a separate discovery area or resources for people curious about SbS. Finally, one of the completers we interviewed for the process evaluation said they were also receiving concurrent face-to-face therapy. Participants for whom this is the case should be flagged in future trials for analysis purposes.

Pilot and feasibility studies aim at identifying potential problems and solutions before running large trials and the lessons learned during this small pilot test are important. It will be necessary to make the intervention much more user-friendly, to enhance recruitment methods (particularly to recruit Syrians, as numbers were so low), as participants complained of technical issues and complexity of sign up. In the two cases of suicidal thoughts that were dealt with by an e-helper and his clinical supervisor, it became apparent that in order for e-helpers to feel more confident with such issues, it will be necessary to ensure more training for e-helpers on suicide case management. Lack of motivation was cited as a barrier for engagement with SbS, so for the next version, we will increase motivational content. Despite an in-depth cultural adaptation process where cultural and contextual relevance was balanced with fidelity to therapeutic content (13), some participants and drop-outs mentioned that the story was not relevant to them and their life circumstances, which could have been a reason for high attrition. Ahead of the next round of testing SbS, we will review the story, making a separate tailored version, more relevant to single users. This would include the characters being younger, working, or studying and will refer to activities more akin to typical activities in an approximately 18–25 age group. The app version of the intervention will be more attractive, engaging, and user-friendly. Evidence indicates that attrition rates are lower in highly interactive e-interventions (5, 6, 27), and although more evidence is needed on the question of participant retention, we envisage that the ease of use, ability to send participants motivational notifications directly to smartphones, and the offline capabilities of an app may reduce attrition rates. If attrition is still high in subsequent testing, we hope to carry out more process evaluation phases to understand why.

## Conclusion

The SbS e-mental health intervention has feasibly resulted in reductions in symptoms of depression and anxiety among those who completed the intervention, though the study was uncontrolled and sample size was very small. Post-intervention assessment completers also indicated a reduction in disability and perceived life problems along with an increase in wellbeing. The quantitative results, as well as the qualitative process evaluation and responses to a client satisfaction questionnaire provide support for the potential of the intervention to help people living in Lebanon, however, it is essential to make some content and procedural adaptations to the app version of the intervention ahead of randomized testing in the coming year. This form of treatment could, in the future, increase capacity to provide care to people living in Lebanon *via* the Internet who would otherwise not have access to evidence-based care.

## Data Availability Statement

The datasets generated for this study are available on request to the corresponding author.

## Ethics Statement

This study was carried out in accordance with the recommendations of the WHO Research Ethics Review Committee and the St Joseph’s University Ethics Committee in Beirut. Written informed consent was provided through the Step-by-Step website at sign-up to the program. All subjects gave written informed consent in accordance with the Declaration of Helsinki. The protocol was approved by the WHO Research Ethics Review Committee and the St Joseph’s University Ethics Committee in Beirut, Lebanon.

## Author Contributions

SW, MH, EvH, KC, and MO designed the intervention, with the support of JA, RC, EZ, and WK for local translation and adaptation. EH and AW were responsible for creating and managing the website. MH, JA, and KC designed and conducted the study with the support of PC, RC, EH, WK, KS, MO, EvH, AW, and EZ. MH, JA, and PC analyzed the data and all other authors contributed to the interpretation of the data. MH and JA wrote the manuscript and all authors carefully reviewed the manuscript and gave final approval of the work to be published.

## Funding

This work was supported and funded by Fondation d’Harcourt, Geneva, Switzerland (Grant No. 64366).

## Conflict of Interest

The authors declare that the research was conducted in the absence of any commercial or financial relationships that could be construed as a potential conflict of interest.

## Abbreviations

SbS, Step-by-Step; MoPH, Ministry of Public Health, Lebanon; PHCC, Primary Health Care Center; WHO, World Health Organization.
