# Supplementary material for: Step-by-Step, an E-Mental Health Intervention for Depression: A Mixed Methods Pilot Study From Lebanon
Source: Front Psychiatry. 2020 Feb 12;10:986. doi: 10.3389/fpsyt.2019.00986 (PMC7034323; doi:10.3389/fpsyt.2019.00986)
Supplement: Supplementary file 1 [file DataSheet_1.docx]

**Supplementary material 1. Process evaluation semi-structured interview guide for participants of the intervention.**

Semi-structured qualitative interviews conducted after the e-mental health intervention pilot intervention

Key informant interviews will last up to one hour, and will follow the following semi-structured interview guide. Informed consent will be obtained immediately prior to interviews (Consent form at the end of this document)). Interviews will be conducted no longer than 6 weeks after the conduct of final outcome assessments. In the case of users, interviews may take place over the phone and thus consent will be taken orally in that case.

All key informant data will be recorded, main points translated, and analyzed following inductive thematic analysis. Findings from this phase of the study will be used to further refine intervention delivery to the local context where required, and to inform the future proposed RCTs of intervention effectiveness.

**Part 1. Interview Guide: Intervention Participants**

1. Greet person. Introduce self, including what organization you are working for. Explain the study following written informed consent process (Consent form at the end of this document)).

Possible additional explanation of semi-structured interview process:

*We would like to ask you some questions about your experience of the internet-based programme that you used, to help us to think about how it could be improved in the future. There are no right or wrong answers to the questions we are going to ask. The interview will be recorded without any identifier to make sure that answers are transcribed accurately; all answers will be reported anonymously to ensure confidentiality. We will be speaking to a number of people, asking everyone the same questions. If you feel unable to answer a question please say and we will move on to the next one.*

1. In note book document date and site of interview, age and gender of interviewee, their position but not their name (i.e. intervention participant, helper, Head of clinic), and initials of interviewers.
2. Begin semi-structured interview:

Record responses and make pertinent notes in the notebook.

Interview process:

- Overall impressions:
  - *1- Please describe your experience of the internet-based programme that you used*
    - Explore positive / negative views through probes.
- Intervention:
  - *2- Describe that we will be making an app for the next phase. Ask the participant to think back to their experience of using the website and ask for suggestions for the app, including features. (any feature or function you would like us to change or add in the app? Any recommendations for colors, etc..)*
  - *3- What did you think of the story? (probe if needed: how did you feel about its relevance, Redundancy, difficulty, Motivational...)*
  - *4- What did you think of the audio exercises? (probe if needed: voice, pace, difficulty, effectiveness)*
    - *What could be improved?*
  - *5- What did you think of the interactive activities?*
    - *What could be improved?*
- Rapport with helper:
  - *6- Please describe how you found working with your helper*
    - Explore positive / negative views through probes.
- 7- How did your family view calls you received or your relationship with your helper?

Explore positive / negative views through probes.

- - *8- How could we improve the contact methods, for example the timings or how we contact users?*
    - Explore further if they are not clear.
- Intervention adherence:
  - *9- Please describe how easy or difficult you found it to complete the five sessions*
    - Explore barriers and facilitators to attendance.
    - Frequency of sessions, length, video vs slides
  - *10- Please describe how you found implementing the skills the helper taught to you in your everyday routine*
    - Explore barriers and facilitators to skills development.
  - *11- How could we encourage users to use SbS as intended e.g. reading the story, watching video, or filling in the activities boxes?*
  - *12- Is there anything else we could do to help users stay motivated?*
    - Explore further if they are not clear.
- Burden of assessments:
  - *13- Please describe how easy or difficult you found doing all the assessments, consenting and registration for this research*

1. Review any written records with the interviewee still present. If anything is not clear ask for clarification and correct written notes as necessary.
2. Ask the interviewee if they have anything to add. Any additional information is added to the interview notes as required.
3. Thank person and leave.
