# Supplementary material for: Step-by-Step, an E-Mental Health Intervention for Depression: A Mixed Methods Pilot Study From Lebanon
Source: Front Psychiatry. 2020 Feb 12;10:986. doi: 10.3389/fpsyt.2019.00986 (PMC7034323; doi:10.3389/fpsyt.2019.00986)
Supplement: Supplementary file 2 [file Table_1.docx]

Supplementary table 1. Characteristics of non-starters versus characteristics of completers.

| **Characteristic** | **Non-starters (n=53)** | **Completers (n=35)** |
| --- | --- | --- |
| Mean age | 27 years old | 28 years old |
| Mean pre-SbS PHQ-8 score | 14.9 | 14.7 |
| Female | 81% | 77% |
| Nationality  Lebanese  Palestinian  Syrian  Other | 89%  4%  4%  4% | 89%  3%  6%  3% |
| Education level  Primary school (3-6y)  Elementary (6-14y)  Secondary school (15-17y)  18+ years | 0  4%  6%  83% | 0  0  9%  91% |
| Occupation status  In work  Student  Homemaker  Unemployed  Other | 40%  26%  15%  13%  4% | 29%  34%  9%  20%  6% |
| Location  Beirut  Outside Beirut  Outside Lebanon  No answer | 42%  45%  13  0 | 40%  57%  0  3% |
| Marital status  Never married  Currently married  Separated/divorced  Widowed | 49%  34%  4%  2% | 66%  26%  9%  0 |
| How did you hear about SbS?  Friend/family  Health worker  Poster/flyer  Internet | 34%  8%  8%  58% | 14%  3%  9%  69% |
